# An International Survey on Taking Up a Career in Cardiovascular Research: Opportunities and Biases toward Would-Be Physician-Scientists

**DOI:** 10.1371/journal.pone.0131900

**Published:** 2015-07-17

**Authors:** Giuseppe Biondi-Zoccai, Enrico Cerrato, Mariangela Peruzzi, Fabrizio D'Ascenzo, Elena De Falco, Isotta Chimenti, Sebastiano Sciarretta, Antonino G. M. Marullo, Elena Cavarretta, Ernesto Greco, Umberto Benedetto, Giulio Pompilio, Javier Escaned, Antonio Abbate, Alain Carpentier, Juan Carlos Chachques, Giacomo Frati

**Affiliations:** 1 Department of Medico-Surgical Sciences and Biotechnologies, Sapienza University of Rome, Latina, Italy; 2 Eleonora Lorillard Spencer Cenci Foundation, Rome, Italy; 3 Division of Cardiology, Rivoli Hospital, Rivoli, Italy; 4 Division of Cardiology, Hospital Clinico San Carlos, Madrid, Spain; 5 Division of Cardiology, University of Turin, Città Della Salute e Delle Scienze San Giovanni Battista, Turin, Italy; 6 Cardiovascular Research Institute, Department of Cell Biology and Molecular Medicine, Rutgers New Jersey Medical School, Newark, NJ, United States of America; 7 Department of Cardiovascular, Respiratory, Nephrological, Anesthesiological, and Geriatric Sciences, Policlinico Umberto I, Sapienza University of Rome, Rome, Italy; 8 Oxford Heart Centre, Oxford University Hospital, Oxford, United Kingdom; 9 Laboratory of Vascular Biology and Regenerative Medicine, Centro Cardiologico Monzino-IRCCS, Milan, Italy; 10 VCU Pauley Heart Center, Virginia Commonwealth University, Richmond, VA, United States of America; 11 Laboratory of Biosurgical Research (Alain Carpentier Foundation), Pompidou Hospital, University Paris Descartes, Paris, France; 12 Department of AngioCardioNeurology, IRCCS Neuromed, Pozzilli, Italy; National Institute of Health, ITALY

## Abstract

**Background:**

Cardiovascular research is the main shaper of clinical evidence underpinning decision making, with its cyclic progression of junior researchers to mature faculty members. Despite efforts at improving cardiovascular research training, several unmet needs persist. We aimed to appraise current perceptions on cardiovascular research training with an international survey.

**Methods and Results:**

We administered a 20-closed-question survey to mentors and mentees belonging to different international institutions. A total of 247 (12%) surveys were available (out of 2,000 invitations). Overall, mentees and mentors were reasonably satisfied with the educational and research resources. Significant differences were found analyzing results according to gender, geographic area, training and full-time researcher status. Specifically, women proved significantly less satisfied than men, disclosed access to fewer resources and less support from mentors (all P<0.05). People working in institutions not located in North America or Northern/Central Europe were significantly less satisfied and disclosed much less support (both P<0.05). Those in training reported limited opportunities for collaboration (P = 0.009), and non-full-time researchers disclosed more limited access to tutors and formal grant writing training (both P<0.05).

**Conclusions:**

Several potential biases appear to be present in the way training in cardiovascular research is provided worldwide, including one against women. If confirmed, these data require proactive measures to decrease discriminations and improve the cardiovascular research training quality.

## Introduction

…Minerva came close up to him in the likeness and with the voice of Mentor. "Telemachus," said she, "if you are made of the same stuff as your father you will be neither fool nor coward henceforward, for Ulysses never broke his word nor left his work half done. If, then, you take after him, your voyage will not be fruitless…

Homer, The Odyssey[1]

Chances in cardiovascular research for fellows, graduate students and for aspiring physician-scientists have never been so exciting as now. The information revolution has revolutionized the flow of biomedical knowledge concerning cardiovascular disease similarly to what the Gutenberg press broadly did in the Renaissance.[2] However, the milieu of cardiovascular research has also become more competitive than in the past.[3] For a young investigator considering the hypothesis of a career in cardiovascular research many challenges have to be overcome, first of all the financial one. In addition, a career combining substantial clinical activities with the concurrent management of a basic, translational or outcome research remains difficult.[4]

Indeed, adequate mentorship and institutional support are key to safely navigate the conundrum represented by concomitant clinical, educational and research activities. Mentorship relies upon the mentor-mentee relationship and it is clearly affected by features of both components.[5] The process of mentoring requires devotion, including generous investment of time, energy, as well as ample resources.[6–9] Yet, it remains poorly studied and evidence of how best to provide mentorship is limited. Given these premises, and aiming at gauging the current stance of both mentees and mentors worldwide toward cardiovascular research training, we designed a survey study.

## Methods

### Survey design

This was an online survey study using fully anonymized data. The survey was designed by an international multidisciplinary team composed of physicians, researchers, academic researchers, residents, fellows and students all involved in the field of cardiovascular medicine. In addition, we created focused groups with residents, fellows and students, and informally interviewed medical school faculty and personnel involved in the clinical education of medical students, to ensure validity and representativeness. Finally, we conducted a dedicated systematic review to acknowledge and build upon prior works on this topic, and also another separate systematic search strategy to identify suitable candidates for the survey (S1 Data).

A total of 20 questions were eventually devised on a dedicated online platform administered by CardioGroup (www.cardiogroup.org) (S2 Data). The survey engine was built following the United State Food and Drug Administration (FDA) recommendations for electronic case report forms, using PHP code language and Oracle MySQL client. Ongoing monitoring for survey accrual and completion was performed, and the survey was closed after having invited 2,000 subjects, followed by descriptive and inferential analyse. This study received the approval of the Department of Medico-Surgical Sciences and Biotechnologies of Sapienza University of Rome, Rome, Italy (S3 Data) and each participant received an invitation-disclosure form before participation (S4 Data).

### Invitation and administration

The survey was distributed locally at the institutions of the international research team, and in addition was sent via email to corresponding authors having published a cardiovascular paper according to a dedicated search strategy. Specifically, email invitations were sent between February 1, 2014 and June 30, 2014. Out of the 2,000 invitations, 251 completed surveys were retrieved. However, 4 surveys had several incomplete entries and were thus excluded from the analysis, yielding a final set of 247 completed questionnaires (response rate of 12%). Survey recipients included people involved in the field of cardiovascular research, including but not limited to full time researchers (which eventually amounted to 70 [28.3%] responders). Response enhancement techniques included mailing lists, multiple mailings and telephone calls. In addition, we encouraged the forwarding of the survey by email to increase participation. The survey was anonymous, without tracking of the IP or other personal features enabling the identification of the respondents. Finally, participation was purely voluntary.

### Statistical Analysis

No specific primary hypothesis or endpoint was outlined. However, the survey was designed to capture several key dimensions of training in cardiovascular research, and also to enable several comparative analyses based on age, sex, geographic area, and training stage of respondents. We did not perform a specific sample size analysis for this work. However, we reasoned that a total of at least 2,000 invitations should have yielded a minimum of 200 completed survey, assuming a 10% response rate. Accordingly, a 200-unit sample would have provided acceptably narrow 95% confidence intervals for inferential analysis (e.g. a 40% positive response to a given question would have yielded a 95% confidence interval ranging from 33% to 47%). Bivariate analyses were performed using the Fisher exact test for categorical variables. Multivariable logistic regression analysis was performed using a backward stepwise selection algorithm (P for removal 0.20), including in the model age, gender, geographic area of origin o respondents, training phase, and full-time researcher status, and reporting odds ratios (OR) with 95% confidence intervals and corresponding P values. Statistical significance was set at the 2-tailed 0.05 level, and P values unadjusted for multiplicity are reported throughout. All computations were performed with SPSS 20 (IBM, Armonk, NY, USA).

## Results

Respondents were mainly men (68.4%), aged between 31 and 40 years (49.8%), and half of them were from Mediterranean countries (57.9%) ("Table 1"). Mentees were 152 (61.5%), those in training were 68 (28.2%), and full-time researchers accounted for 70 (28.3%) subjects (Table 2). Additional analyses are disclosed in S1–S5 Tables.

**Table 1:** Characteristics of participants to the survey.

Features	Participants (N = 247)
Female gender	78 (31.6%)
Age	
≤30 years	83 (33.6%)
31–40 years	123 (49.8%)
>40 years	41 (16.6%)
Geographic area of origin	
North America	13 (5.3%)
Central and South America	15 (6.1%)
Northern and Continental Europe	66 (26.7%)
Mediterranean countries	143 (57.9%)
Asia and Pacific	10 (5.4%)
Geographic area of institution	
North America	23 (9.3%)
Central and South America	10 (4.1%)
Northern and Continental Europe	73 (29.6%)
Mediterranean countries	140 (56.7%)
Asia and Pacific	1 (0.4%)
Current position	
Undergraduate student	3 (1.2%)
Graduate or post-graduate student	25 (10.2%)
Assistant Professor	20 (8.1%)
Associate Professor	5 (2.0%)
Professor	20 (8.1%)
Resident/Fellow	124 (50.2%)
Consultant	34 (13.8%)

**Table 2:** Survey results: overall and distinguishing mentees and mentors.

Question	Mentees (152)	Mentors (N = 95)	Total (N = 247)	P
How many potential areas/fields of research concerning cardiovascular sciences did your institution offer?				0.091
1–2	34 (22.4%)	16 (16.8%)	50 (20.2%)	
3–4	54 (35.5%)	26 (27.4%)	80 (32.4%)	
5–6	32 (21.1%)	19 (20.0%)	51 (20.6%)	
>6	31 (20.4%)	34 (35.8%)	65 (26.3%)	
The field of research concerning cardiovascular sciences you have pursued was your first preference? Yes	98 (64.5%)	65 (68.4%)	163 (68.4%)	0.804
How many times in a week is the tutor available for consultation?				0.272
1–2	58 (38.2%)	40 (42.1%)	98 (39.7%)	
3–4	45 (29.6%)	28 (29.5%)	73 (29.6%)	
5–6	19 (12.5%)	7 (7.4%)	26 (10.5%)	
>6	8 (5.3%)	10 (10.5%)	18 (7.3%)	
I have not yet made up my mind on any specific research project/No answer	21 (13.8%)	8 (8.4%)	29 (11.7%)	
How many potential tutors are available in your institution in this specific area you would like to pursue?				0.006
0	11 (7.2%)	3 (3.2%)	14 (5.7%)	
1	33 (21.7%)	18 (18.9%)	51 (20.6%)	
2	32 (21.1%)	16 (16.8%)	48 (19.4%)	
>2	69 (45.4%)	42 (44.2%)	111 (44.9%)	
Not applicable/No answer	5 (3.3%)	16 (16.8%)	21 (8.5%)	
Did the tutor routinely schedule scientific meetings and/or journal clubs? Yes	76 (50.0%)	41 (43.2%)	117 (47.4%)	0.500
Did the tutor set up a hierarchical structure in order to assure a tutorial program to fellows? Yes	85 (55.9%)	49 (51.6%)	134 (54.3%)	0.797
Do the scientists/researchers which are colleagues of the tutor collaborate to train the fellows? Yes	108 (71.1%)	66 (69.5%)	174 (70.4%)	0.804
Is it an exciting and pleasurable place to work? Yes	112 (73.7%)	68 (71.6%)	180 (72.9%)	0.746
Do tutors treat fellows sensibly and professionally? Yes	117 (77.0%)	62 (65.3%)	179 (72.5%)	0.225
Has each fellow an adequate working space with fully available equipment and supplies? Yes	77 (50.7%)	42 (44.2%)	119 (48.2%)	0.168
Is there opportunity to establish collaborations with other research groups? Yes	112 (73.7%)	83 (87.4%)	195 (78.9%)	0.058
Can the tutor send fellows abroad for training? Yes	103 (67.8%)	65 (68.4%)	168 (68.0%)	0.778
What would be your geographic region of choice to temporary continue your training?				0.223
North America	49 (32.2%)	37 (38.9%)	86 (34.8%)	
Central and South America	0	1 (1.1%)	1 (0.4%)	
Northern and Continental Europe	59 (38.8%)	26 (27.4%)	85 (34.4%)	
Mediterranean countries	43 (28.3%)	29 (30.5%)	72 (29.1%)	
Asia and Pacific	1 (0.7%)	2 (2.1%)	3 (1.2%)	
Has the tutor the opportunity to provide scholarship to fellows? Yes	68 (44.7%)	51 (53.7%)	119 (48.2%)	0.114
Is the tutor willing to foster the fellow independence?	113 (74.3%)	71 (74.7%)	184 (74.5%)	0.777
Does the tutor train fellows in writing scholarly papers? Yes	79 (7.9%)	59 (62.1%)	138 (55.9%)	0.376
Does the tutor train fellows in writing research grants? Yes	51 (33.6%)	39 (41.1%)	90 (36.4%)	0.251
Does the tutor really help fellows in finding an academic position or an appropriate professional employment? Yes	72 (47.4%)	48 (50.5%)	120 (48.6%)	0.880
If you had to do it all over again, would you choose to pursue research/clinical training in this same institution? Yes	104 (68.4%)	64 (67.4%)	168 (68.0%)	0.645

**Fig 1:**
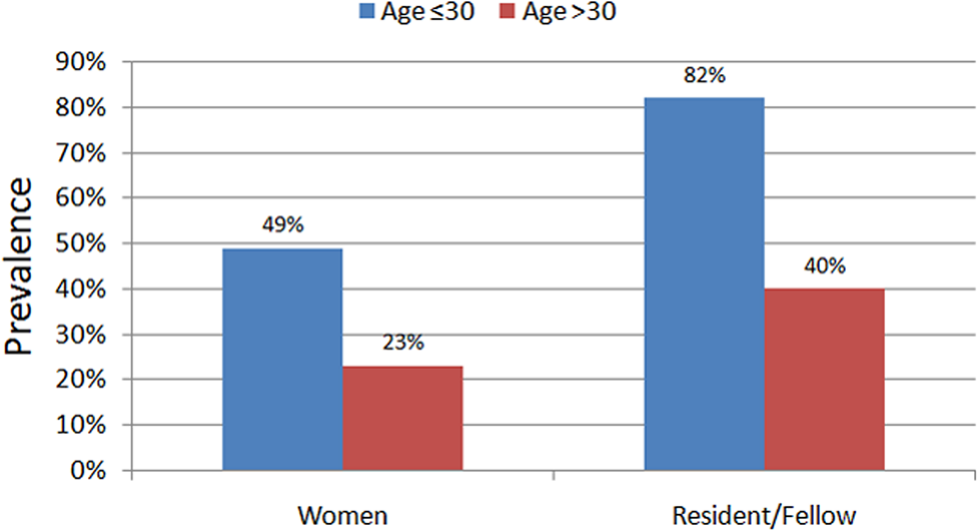
Key differences in replies according to age of respondents.

In particular, age was not associated with any significant difference in replies (all P>0.05) (S1 and S5 Tables). Conversely, several gender-related differences were identified (S1 and S5 Tables; Fig 1). In particular, bivariate and multivariable analyses showed that female respondents were less satisfied with their career path, reported less support from tutors, and disclosed less commonly the possibility to pursue their preferred field of research (all P<0.05).

**Fig 2:**
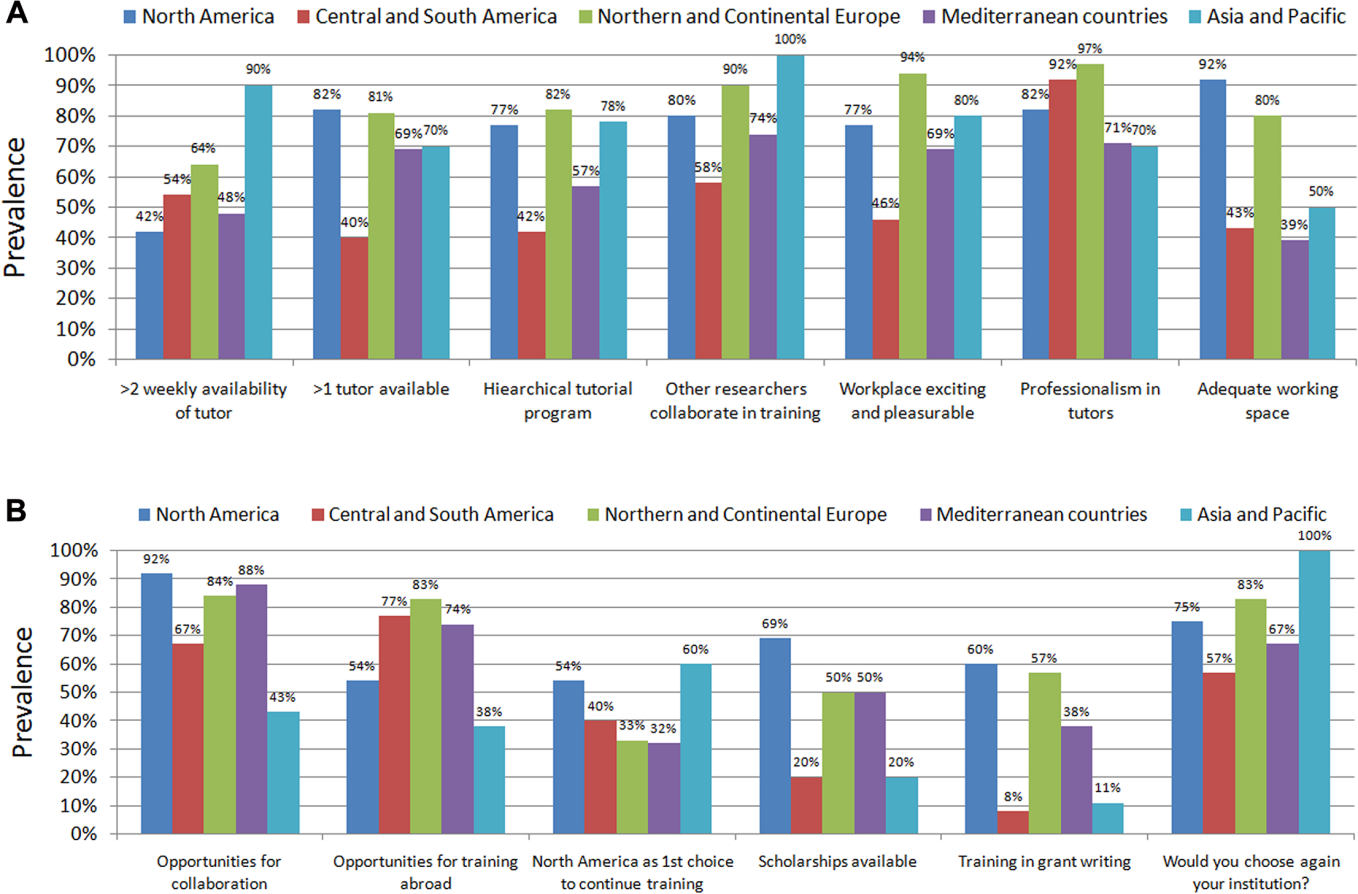
Key differences in replies according to geographic area of origin of respondents (panel A and panel B).

Geographic area of origin was also associated at bivariate and multivariable analysis with a number of significant differences (S2 and S5 Tables). In particular, those who originated from North America or Northern/Continental Europe disclosed more often that their institution was a proactive one, with adequate facilities, and that tutors were helpful in finding an appropriate appointment (all P<0.05) (Fig 2A). Accordingly, they were more satisfied than other respondents with their career path, and were more likely to choose the same geographic area to pursue further training (both P<0.05) (Fig 2B).

**Fig 3:**
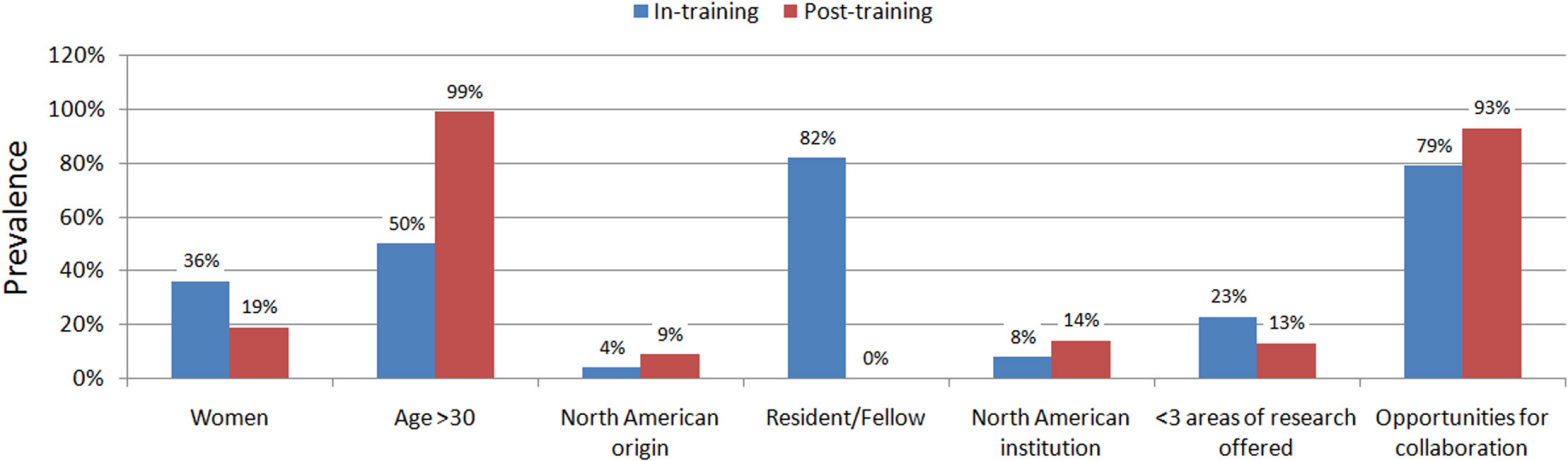
Key differences in replies according to phase of training of respondents.

The only feature which appeared statistically significant between respondents in training and post-training was that those belonging to the former group acknowledge less opportunities than those post-training (P = 0.009) (S3 and S5 Tables; Fig 3). Comparison of those working full-time in research versus those with a lower commitment highlighted that full-time researcher status was associated a higher likelihood of replying that tutors were often available to mentees and that they provided formal training in grant writing (both P<0.05) (S4 and S5 Tables). Conversely, detailed bivariate analysis for mentee versus mentor status did not disclose any significant difference in replies (with the notable exclusion of an obvious lower likelihood of having defined already a preferred field of research among mentees [P = 0.006]) (Table 2).

## Discussion

This work build upon other surveys on training in cardiovascular research or practice have recently been published.[3,9–12]. In particular, our results highlighting a potential bias in cardiovascular research training towards women in unprecedented.

Gender may indeed play a complex role on the interaction between institutions, mentors, and trainees.[9,13] The delicate issue concerning a possible differential attitude of mentors towards male or female mentees has been evidenced by the differentially perceived frequency of mentors availability for consultations. Although this may also arise from highest expectations and demands from female trainees compared to their male colleagues, nevertheless it can be considered as a sign of lower satisfaction for early career women in the field. Moreover, different perspectives among men and women towards the quality of the research mentorship they are offered have not been reported before.[10,11,14] Thus, caution must be exercised in interpreting and using them for policy making, until they can be confirmed independently.[15] However, there is already evidence that men are more satisfied of their clinical training in general surgery and consider more rarely to leave it, with women reporting less commonly support from the program, less access to faculty members when having difficulties, less respect from the attending, and less solidarity from peers.[16] Similar findings have been reported for allied health care personnel.[17] In addition, supporting data have been provided in a careful analysis of authors and peer reviewers of a scholarly journal.[18] Finally, sex-related bias may operate at several levels, even after a faculty appointment, with women obtaining less opportunity to promotion or being relegated to non-leadership positions.[19]

The other significant predictors of replies are also important, in particular the one depending on geographic area of origin, and those correlated with training phase and full-time researcher status. It is clear that there are major disparities in the quality with which cardiovascular research and training is provided in different countries. Moreover, it is not unexpected to recognize discrepancies in the responses on cardiovascular research training when comparing those post-training and/or working as full-time researchers versus those in training or doing research only as part-time occupation. Yet, these differences should as well call for reasonable but integrated efforts at ameliorating the current situation including the well-known brain drain phenomenon.

Indeed, how can things be improved? First, diversity should be encouraged and open access to researcher networks and facilities should be provided. Training should include in the core curriculum grant writing. In addition, flexible programs may be set already at the undergraduate level to improve confidence, satisfaction, productivity and collaboration.[20] Other measures can also be implemented. However, the first step in addressing existing bias is recognizing them and quantifying them appropriately.

This work has several limitations, which include the observational cross-sectional design, reliance only on closed-ended questions, and risk of false answers. In addition, the international features of our survey possibly limit its validity for country-specific or region-specific situations. In addition, we invited both mentees and mentors, limiting our chance to pose position-specific questions. However, this choice was pursued as it would have enabled comparison of perspectives stemming from these two groups of people. In addition, sample size and validity could have been boosted if two separate surveys had been performed. Most importantly, however, every mentor has been a mentee in the past, and we wish most mentees to become mentors in the future.[21] Thus, it is not so easy to cross a borderline and distinguish the two groups and some of their opinions may be interpreted at both pooled and stratified level. In addition, we did not inquire in detail on number of publications or grants accepted, to verify the real academic standing of the participants. Further surveys are thus clearly warranted to address the crucial mentee versus mentor comparison, as well as the correlation between publications and grants with responses to surveys similar to our own. Finally, we performed several bivariate analyses on top of our descriptive overall analysis. Accordingly, the risk of multiplicity and type I error inflation in such unadjusted analyses should be carefully borne in mind.

## Supporting Information

S1 Table Survey results according to age and sex of respondents. (DOC)

S2 Table Survey results according to geographic area of origin of respondents. (DOC)

S3 Table Survey results according to phase of training of respondents. (DOC)

S4 Table Survey results according to full-time researcher status. (DOC)

S5 Table Multivariable analysis for selected responses. (DOC)

S1 Data Search strategy. (DOC)

S2 Data Questionnaire. (DOC)

S3 Data Institutional approval. (PDF)

S4 Data Invitation letter. (DOC)
