# Supplementary material for: An International Survey on Taking Up a Career in Cardiovascular Research: Opportunities and Biases toward Would-Be Physician-Scientists
Source: PLoS One. 2015 Jul 17;10(7):e0131900. doi: 10.1371/journal.pone.0131900 (PMC4506064; doi:10.1371/journal.pone.0131900)
Supplement: S1 Table — (DOC) [file pone.0131900.s001.doc]

**Table S1.** Survey results according to age and sex of respondents.

|  | **Age ≤30 (N=82)** | **Age >30 (N=162)** | **P** | **Male (N=167)** | **Female (N=78)** | **P** |
| --- | --- | --- | --- | --- | --- | --- |
| How many potential areas/fields of research concerning cardiovascular sciences did your institution offer? |  |  | 0.638 |  |  | 0.045 |
| 1-2 | 20 (24.4%) | 29 (17.9%) |  | 31 (18.6%) | 19 (24.4%) |  |
| 3-4 | 27 (32.9%) | 53 (32.7%) |  | 56 (33.5%) | 24 (30.8%) |  |
| 5-6 | 15 (18.3%) | 36 (22.2%) |  | 29 (17.4%) | 22 (28.2%) |  |
| >6 | 20 (24.4%) | 44 (27.2%) |  | 51 (30.5%) | 13 (16.7%) |  |
| The field of research concerning cardiovascular sciences you have pursued was your first preference? |  |  | 0.336 |  |  | <0.001 |
| Yes | 51 (71.8%) | 111 (75.5%) |  | 124 (82.7%) | 38 (55.1%) |  |
| No | 20 (28.2%) | 36 (24.5%) |  | 26 (17.3%) | 31 (44.9%) |  |
| How many times in a week is the tutor available for consultation? |  |  | 0.091 |  |  | 0.259 |
| 1-2 | 36 (51.4%) | 61 (42.4%) |  | 63 (41.5%) | 34 (54.8%) |  |
| 3-4 | 21 (30.0%) | 52 (36.1%) |  | 53 (34.9%) | 20 (32.3%) |  |
| 5-6 | 11 (15.7%) | 15 (10.4%) |  | 21 (13.8%) | 5 (8.1%) |  |
| >6 | 2 (2.9%) | 16 (11.1%) |  | 15 (9.9%) | 3 (4.8%) |  |
| How many potential tutors are available in your institution in this specific area you would like to pursue? |  |  | 0.273 |  |  | 0.055 |
| 0 | 6 (7.8%) | 8 (5.5%) |  | 7 (4.6%) | 7 (10.0%) |  |
| 1 | 21 (27.3%) | 30 (20.7%) |  | 35 (22.9%) | 16 (22.9%) |  |
| 2 | 11 (14.3%) | 36 (24.1%) |  | 27 (17.7%) | 20 (28.6%) |  |
| >2 | 39 (50.7%) | 72 (49.7%) |  | 84 (54.9%) | 27 (38.6%) |  |
| Did the tutor routinely schedule scientific meetings and/or journal clubs? |  |  | 0.667 |  |  | 0.384 |
| Yes | 37 (51.4%) | 80 (54.8%) |  | 83 (55.7%) | 34 (48.6%) |  |
| No | 35 (48.6%) | 66 (45.2%) |  | 66 (44.3%) | 36 (51.4%) |  |
| Did the tutor set up a hierarchical structure in order to assure a tutorial program to fellows? |  |  | 0.882 |  |  | 1.0 |
| Yes | 45 (63.4%) | 89 (61.8%) |  | 90 (62.1%) | 44 (62.0%) |  |
| No | 26 (36.6%) | 55 (38.2%) |  | 55 (37.9%) | 27 (38.0%) |  |
| Do the scientists/researchers which are colleagues of the tutor collaborate to train the fellows? |  |  | 0.163 |  |  | 0.109 |
| Yes | 55 (73.3%) | 118 (81.9%) |  | 125 (81.7%) | 48 (71.6%) |  |
| No | 20 (26.7%) | 26 (18.1%) |  | 28 (18.3%) | 19 (28.4%) |  |
| Is it an exciting and pleasurable place to work? |  |  | 0.341 |  |  | 0.335 |
| Yes | 57 (71.3%) | 123 (77.4%) |  | 127 (77.0%) | 53 (70.7%) |  |
| No | 23 (28.8%) | 36 (22.6%) |  | 38 (23.0%) | 22 (29.3%) |  |
| Do tutors treat fellows sensibly and professionally? |  |  | 1.0 |  |  | 0.213 |
| Yes | 63 (79.8%) | 116 (80.0%) |  | 127 (81.9% | 52 (74.3%) |  |
| No | 16 (20.2%) | 29 (20.0%) |  | 28 (18.1%) | 18 (25.7%) |  |
| Has each fellow an adequate working space with fully available equipment and supplies? |  |  | 0.569 |  |  | 0.476 |
| Yes | 41 (56.2%) | 78 (51.7%) |  | 84 (54.6%) | 35 (49.3%) |  |
| No | 32 (43.8%) | 73 (48.3%) |  | 70 (45.4%) | 36 (50.7%) |  |
| Is there opportunity to establish collaborations with other research groups? |  |  | 0.848 |  |  | 0.846 |
| Yes | 64 (85.3%) | 130 (83.9%) |  | 134 (84.8%) | 61 (83.6%) |  |
| No | 11 (14.7%) | 25 (16.1%) |  | 24 (15.2%) | 12 (16.4%) |  |
| Can the tutor send fellows abroad for training? |  |  | 0.733 |  |  | 0.863 |
| Yes | 56 (75.7%) | 111 (78.2%) |  | 113 (77.9%) | 55 (76.4%) |  |
| No | 18 (24.3%) | 31 (21.8%) |  | 32 (22.1%) | 17 (23.6%) |  |
| What would be your geographic region of choice to temporary continue your training? |  |  | 1.0 |  |  | 0.177 |
| North America | 29 (34.9%) | 57 (35.2%) |  | 65 (38.7%) | 21 (26.9%) |  |
| Central and South America | 0 | 1 (0.6%) |  | 1 (0.6%) | 0 |  |
| Northern and Continental Europe | 29 (34.9%) | 55 (34.0%) |  | 53 (31.6%) | 32 (41.0%) |  |
| Mediterranean countries | 24 (28.9%) | 47 (29.0%) |  | 48 (28.6%) | 23 (29.5%) |  |
| Asia and Pacific | 1 (1.2%) | 2 (1.2%) |  | 1 (0.6%) | 2 (2.6%) |  |
| Has the tutor the opportunity to provide scholarship to fellows? |  |  | 0.886 |  |  | 0.661 |
| Yes | 41 (55.4%) | 78 (53.8%) |  | 84 (55.3%) | 35 (51.5%) |  |
| No | 33 (44.6%) | 67 (46.2%) |  | 68 (44.7%) | 33 (48.5%) |  |
| Is the tutor willing to foster the fellow independence? |  |  | 0.695 |  |  | 0.166 |
| Yes | 62 (82.7%) | 122 (85.3%) |  | 129 (86.6%) | 55 (78.6%) |  |
| No | 13 (17.3%) | 21 (14.7%) |  | 20 (13.4%) | 15 (21.4%) |  |
| Does the tutor train fellows in writing scholarly papers? |  |  | 0.568 |  |  | 0.312 |
| Yes | 45 (58.4%) | 93 (62.4%) |  | 97 (63.0%) | 41 (55.4%) |  |
| No | 32 (41.6%) | 56 (37.6%) |  | 56 (37.0%) | 33 (44.6%) |  |
| Does the tutor train fellows in writing research grants? |  |  | 1.0 |  |  | 1.0 |
| Yes | 32 (41.0%) | 58 (40.9%) |  | 60 (40.8%) | 30 (41.1%) |  |
| No | 46 (59.0%) | 84 (59.2%) |  | 87 (59.2%) | 43 (58.9%) |  |
| Does the tutor really help fellows in finding an academic position or an appropriate professional employment? |  |  | 0.546 |  |  | 0.034 |
| Yes | 41 (62.1%) | 79 (57.3%) |  | 89 (63.6%) | 31 (47.7%) |  |
| No | 25 (37.9%) | 59 (42.8%) |  | 51 (36.4%) | 34 (52.3%) |  |
| If you had to do it all over again, would you choose to pursue research/clinical training in this same institution? |  |  | 0.442 |  |  | 0.001 |
| Yes | 55 (69.6%) | 113 (74.3%) |  | 125 (79.1%) | 43 (58.1%) |  |
| No | 24 (30.4%) | 39 (25.7%) |  | 33 (20.9%) | 31 (41.9%) |  |
