# Supplementary material for: An International Survey on Taking Up a Career in Cardiovascular Research: Opportunities and Biases toward Would-Be Physician-Scientists
Source: PLoS One. 2015 Jul 17;10(7):e0131900. doi: 10.1371/journal.pone.0131900 (PMC4506064; doi:10.1371/journal.pone.0131900)
Supplement: S2 Table — (DOC) [file pone.0131900.s002.doc]

**Table S2.** Survey results according to geographic area of origin of respondents.

|  | **North America**  **(N=13)** | **Central and South America (N=15)** | **Northern and Continental Europe (N=66)** | **Mediterranean countries (N=143)** | **Asia and Pacific (N=10)** | **P** |
| --- | --- | --- | --- | --- | --- | --- |
| How many potential areas/fields of research concerning cardiovascular sciences did your institution offer? |  |  |  |  |  | 0.072 |
| 1-2 | 2 (15.4%) | 6 (40.0%) | 11 (16.9%) | 29 (20.3%) | 2 (20.0%) |  |
| 3-4 | 4 (30.8%) | 4 (26.7%) | 20 (30.8%) | 46 (32.2%) | 6 (60.0%) |  |
| 5-6 | 2 (15.4%) | 2 (13.3%) | 8 (12.3%) | 38 (26.6%) | 1 (10.0%) |  |
| >6 | 5 (38.5%) | 3 (20.0%) | 26 (40.0%) | 30 (21.0%) | 1 (10.0%) |  |
| The field of research concerning cardiovascular sciences you have pursued was your first preference? |  |  |  |  |  | 0.128 |
| Yes | 2 (15.4%) | 5 (41.7%) | 10 (17.0%) | 39 (30.7%) | 1 (11.1%) |  |
| No | 11 (84.6%) | 7 (58.3%) | 49 (83.1%) | 88 (69.3%) | 8 (88.9%) |  |
| How many times in a week is the tutor available for consultation? |  |  |  |  |  | 0.026 |
| 1-2 | 7 (58.3%) | 5 (45.5%) | 20 (35.7%) | 65 (51.6%) | 1 (10.0%) |  |
| 3-4 | 3 (25.0%) | 2 (18.2%) | 22 (39.3%) | 39 (31.0%) | 7 (70.0%) |  |
| 5-6 | 1 (8.3%) | 4 (36.4%) | 5 (8.9%) | 15 (11.9%) | 1 (10.0%) |  |
| >6 | 1 (8.3%) | 0 | 9 (16.1%) | 7 (5.6%) | 1 (10.0%) |  |
| How many potential tutors are available in your institution in this specific area you would like to pursue? |  |  |  |  |  | 0.055 |
| 0 | 1 (9.1%) | 3 (20.0%) | 2 (3.1%) | 8 (6.5%) | 0 |  |
| 1 | 1 (9.1%) | 6 (40.0%) | 10 (15.6%) | 31 (25.0%) | 3 (30.0%) |  |
| 2 | 3 (27.3%) | 1 (6.7%) | 16 (25.0%) | 23 (18.6%) | 5 (50.0%) |  |
| >2 | 6 (54.6%) | 5 (33.3%) | 36 (56.3%) | 62 (50.0%) | 2 (20.0%) |  |
| Did the tutor routinely schedule scientific meetings and/or journal clubs? |  |  |  |  |  | 0.108 |
| Yes | 8 (72.7%) | 6 (42.9%) | 34 (61.8%) | 62 (47.7%) | 7 (77.8%) |  |
| No | 3 (27.3%) | 8 (57.1%) | 21 (38.2%) | 68 (52.3%) | 2 (22.2%) |  |
| Did the tutor set up a hierarchical structure in order to assure a tutorial program to fellows? |  |  |  |  |  | 0.104 |
| Yes | 7 (63.6%) | 5 (41.7%) | 42 (73.7%) | 73 (57.0%) | 7 (77.8%) |  |
| No | 4 (36.4%) | 7 (58.3%) | 15 (26.3%) | 55 (43.0%) | 2 (22.2%) |  |
| Do the scientists/researchers which are colleagues of the tutor collaborate to train the fellows? |  |  |  |  |  | 0.015 |
| Yes | 8 (80.0%) | 7 (58.3%) | 53 (89.8%) | 97 (74.1%) | 9 (100%) |  |
| No | 2 (20.0%) | 5 (41.7%) | 6 (10.2%) | 34 (26.0%) | 0 |  |
| Is it an exciting and pleasurable place to work? |  |  |  |  |  | <0.001 |
| Yes | 10 (76.9%) | 6 (46.2%) | 60 (93.8%) | 96 (68.6%) | 8 (80.0%) |  |
| No | 3 (23.1%) | 7 (53.8%) | 4 (6.3%) | 44 (31.4%) | 2 (20.0%) |  |
| Do tutors treat fellows sensibly and professionally? |  |  |  |  |  | <0.001 |
| Yes | 9 (81.8%) | 12 (92.3%) | 56 (96.6%) | 95 (71.4%) | 7 (70.0%) |  |
| No | 2 (18.2%) | 1 (7.7%) | 2 (3.5%) | 38 (28.6%) | 3 (30.0%) |  |
| Has each fellow an adequate working space with fully available equipment and supplies? |  |  |  |  |  | <0.001 |
| Yes | 11 (91.7%) | 6 (42.9%) | 47 (79.7%) | 50 (38.5%) | 5 (50.0%) |  |
| No | 1 (8.3%) | 8 (57.1%) | 12 (20.3%) | 80 (61.5%) | 5 (50.0%) |  |
| Is there opportunity to establish collaborations with other research groups? |  |  |  |  |  | 0.011 |
| Yes | 12 (92.3%) | 10 (66.7%) | 52 (83.9%) | 118 (88.1%) | 3 (42.9%) |  |
| No | 1 (7.7%) | 5 (33.3%) | 10 (16.1%) | 16 (11.9%) | 4 (57.1%) |  |
| Can the tutor send fellows abroad for training? |  |  |  |  |  | 0.029 |
| Yes | 7 (53.9%) | 10 (76.9%) | 49 (83.1%) | 97 (74.1%) | 3 (37.5%) |  |
| No | 6 (46.2%) | 3 (23.1%) | 10 (17.0%) | 34 (25.9%) | 5 (62.5%) |  |
| What would be your geographic region of choice to temporary continue your training? |  |  |  |  |  | 0.002 |
| North America | 7 (53.9%) | 6 (40.0%) | 22 (33.3%) | 45 (31.5%) | 6 (60.0%) |  |
| Central and South America | 0 | 0 | 0 | 1 (0.7%) | 0 |  |
| Northern and Continental Europe | 3 (23.1%) | 1 (6.7%) | 35 (53.0%) | 42 (29.4%) | 4 (40.0%) |  |
| Mediterranean countries | 2 (15.4%) | 8 (53.3%) | 9 (13.6%) | 53 (37.1%) | 0 |  |
| Asia and Pacific | 1 (7.7%) | 0 | 0 | 2 (1.4%) | 0 |  |
| Has the tutor the opportunity to provide scholarship to fellows? |  |  |  |  |  | 0.021 |
| Yes | 9 (69.2%) | 3 (20.0%) | 33 (50.0%) | 72 (50.3%) | 2 (20.0%) |  |
| No | 2 (15.4%) | 8 (53.3%) | 25 (37.9%) | 58 (40.6%) | 8 (80.0%) |  |
| Is the tutor willing to foster the fellow independence? |  |  |  |  |  | 0.840 |
| Yes | 9 (81.8%) | 12 (92.3%) | 47 (87.0%) | 108 (82.4%) | 8 (80.0%) |  |
| No | 2 (18.2%) | 1 (7.7%) | 7 (13.0%) | 23 (17.6%) | 2 (20.0%) |  |
| Does the tutor train fellows in writing scholarly papers? |  |  |  |  |  | 0.221 |
| Yes | 7 (63.6%) | 6 (46.2%) | 42 (72.4%) | 78 (57.8%) | 5 (50.0%) |  |
| No | 4 (36.4%) | 7 (53.9%) | 16 (27.6%) | 57 (42.2%) | 5 (50.0%) |  |
| Does the tutor train fellows in writing research grants? |  |  |  |  |  | 0.001 |
| Yes | 6 (60.0%) | 1 (7.7%) | 34 (56.7%) | 48 (37.5%) | 1 (11.1%) |  |
| No | 4 (40.0%) | 12 (92.3%) | 26 (43.3%) | 80 (62.5%) | 8 (89.9%) |  |
| Does the tutor really help fellows in finding an academic position or an appropriate professional employment? |  |  |  |  |  | 0282 |
| Yes | 7 (70.0%) | 10 (76.9%) | 34 (61.8%) | 66 (55.9%) | 3 (33.3%) |  |
| No | 3 (30.0%) | 3 (23.1%) | 21 (38.2%) | 52 (44.1%) | 6 (66.7%) |  |
| If you had to do it all over again, would you choose to pursue research/clinical training in this same institution? |  |  |  |  |  | 0.021 |
| Yes | 9 (75.0%) | 8 (57.1%) | 49 (83.1%) | 92 (67.2%) | 10 (100%) |  |
| No | 3 (25.0%) | 6 (42.9%) | 10 (17.0%) | 45 (32.9%) | 0 |  |
