# Supplementary material for: An International Survey on Taking Up a Career in Cardiovascular Research: Opportunities and Biases toward Would-Be Physician-Scientists
Source: PLoS One. 2015 Jul 17;10(7):e0131900. doi: 10.1371/journal.pone.0131900 (PMC4506064; doi:10.1371/journal.pone.0131900)
Supplement: S3 Table — (DOC) [file pone.0131900.s003.doc]

**Table S3.** Survey results according to phase of training of respondents.

|  | **Training (N=68)** | **Post-training (N=79)** | **P** |
| --- | --- | --- | --- |
| How many potential areas/fields of research concerning cardiovascular sciences did your institution offer? |  |  | 0.029 |
| 1-2 | 34 (22.5%) | 10 (12.7%) |  |
| 3-4 | 54 (35.8%) | 23 (29.1%) |  |
| 5-6 | 32 (21.2%) | 16 (20.3%) |  |
| >6 | 31 (20.5%) | 30 (38.0%) |  |
| The field of research concerning cardiovascular sciences you have pursued was your first preference? |  |  | 0.248 |
| Yes | 98 (85.2%) | 55 (90.2%) |  |
| No | 17 (14.8%) | 6 (9.8%) |  |
| How many times in a week is the tutor available for consultation? |  |  | 0.274 |
| 1-2 | 58 (44.6%) | 31 (43.1%) |  |
| 3-4 | 45 (34.6%) | 24 (33.3%) |  |
| 5-6 | 19 (14.6%) | 7 (9.7%) |  |
| >6 | 8 (6.2%) | 10 (13.9%) |  |
| How many potential tutors are available in your institution in this specific area you would like to pursue? |  |  | 0.663 |
| 0 | 11 (7.6%) | 3 (4.6%) |  |
| 1 | 33 (22.8%) | 13 (20.0%) |  |
| 2 | 32 (22.1%) | 12 (18.5%) |  |
| >2 | 69 (47.6%) | 37 (56.9%) |  |
| Did the tutor routinely schedule scientific meetings and/or journal clubs? |  |  | 0.466 |
| Yes | 76 (55.5%) | 35 (50.0%) |  |
| No | 61 (44.5%) | 35 (50.0%) |  |
| Did the tutor set up a hierarchical structure in order to assure a tutorial program to fellows? |  |  | 0.761 |
| Yes | 85 (63.4%) | 42 (60.9%) |  |
| No | 49 (36.6%) | 27 (39.1%) |  |
| Do the scientists/researchers which are colleagues of the tutor collaborate to train the fellows? |  |  | 1.0 |
| Yes | 108 (80.0%) | 57 (79.2%) |  |
| No | 27 (20.0%) | 15 (20.8%) |  |
| Is it an exciting and pleasurable place to work? |  |  | 0.868 |
| Yes | 112 (76.2%) | 61 (78.2%) |  |
| No | 35 (23.8%) | 17 (21.8%) |  |
| Do tutors treat fellows sensibly and professionally? |  |  | 0.102 |
| Yes | 117 (83.6%) | 53 (73.6%) |  |
| No | 23 (16.4%) | 19 (26.4%) |  |
| Has each fellow an adequate working space with fully available equipment and supplies? |  |  | 0.253 |
| Yes | 77 (57.0%) | 37 (48.7%) |  |
| No | 58 (43.0%) | 39 (51.3%) |  |
| Is there opportunity to establish collaborations with other research groups? |  |  | 0.010 |
| Yes | 112 (79.4%) | 70 (93.3%) |  |
| No | 29 (20.6%) | 5 (6.7%) |  |
| Can the tutor send fellows abroad for training? |  |  | 0.601 |
| Yes | 103 (79.9%) | 57 (80.3%) |  |
| No | 31 (23.1%) | 14 (19.7%) |  |
| What would be your geographic region of choice to temporary continue your training? |  |  | 0.050 |
| North America | 49 (32.2%) | 34 (43.0%) |  |
| Central and South America | 0 | 1 (1.3%) |  |
| Northern and Continental Europe | 59 (38.8%) | 19 (24.1%) |  |
| Mediterranean countries | 43 (28.3%) | 23 (29.1%) |  |
| Asia and Pacific | 1 (0.7%) | 2 (2.5%) |  |
| Has the tutor the opportunity to provide scholarship to fellows? |  |  | 0.107 |
| Yes | 68 (50.0%) | 45 (62.5%) |  |
| No | 66 (50.0%) | 27 (37.5%) |  |
| Is the tutor willing to foster the fellow independence? |  |  | 0.292 |
| Yes | 113 (83.7%) | 64 (90.1%) |  |
| No | 22 (16.3%) | 7 (7.9%) |  |
| Does the tutor train fellows in writing scholarly papers? |  |  | 0.190 |
| Yes | 79 (57.7%) | 51 (67.1%) |  |
| No | 58 (42.3%) | 25 (32.9%) |  |
| Does the tutor train fellows in writing research grants? |  |  | 0.038 |
| Yes | 51 (37.5%) | 37 (52.9%) |  |
| No | 85 (62.5%) | 33 (47.1%) |  |
| Does the tutor really help fellows in finding an academic position or an appropriate professional employment? |  |  | 0.878 |
| Yes | 72 (58.1%) | 40 (59.7%) |  |
| No | 52 (41.9%) | 27 (40.3%) |  |
| If you had to do it all over again, would you choose to pursue research/clinical training in this same institution? |  |  | 1.0 |
| Yes | 104 (72.7%) | 56 (73.7%) |  |
| No | 39 (27.3%) | 20 (26.3%) |  |
