# Supplementary material for: An International Survey on Taking Up a Career in Cardiovascular Research: Opportunities and Biases toward Would-Be Physician-Scientists
Source: PLoS One. 2015 Jul 17;10(7):e0131900. doi: 10.1371/journal.pone.0131900 (PMC4506064; doi:10.1371/journal.pone.0131900)
Supplement: S4 Table — (DOC) [file pone.0131900.s004.doc]

**Table S4.** Survey results according to full-time researcher status.

|  | **Non-full-time researcher (N=177)** | **Full-time researcher (N=70)** | **P** |
| --- | --- | --- | --- |
| How many potential areas/fields of research concerning cardiovascular sciences did your institution offer? |  |  | 0.006 |
| 1-2 | 45 (25.4%) | 5 (7.1%) |  |
| 3-4 | 59 (33.3%) | 21 (30.0%) |  |
| 5-6 | 33 (18.6%) | 18 (25.7%) |  |
| >6 | 39 (22.0%) | 26 (37.1%) |  |
| The field of research concerning cardiovascular sciences you have pursued was your first preference? |  |  | 0.194 |
| Yes | 112 (63.3%) | 51 (72.9%) |  |
| No | 41 (23.2%) | 16 (22.9%) |  |
| How many times in a week is the tutor available for consultation? |  |  | 0.005 |
| 1-2 | 73 (41.2%) | 25 (35.7%) |  |
| 3-4 | 54 (30.5%) | 19 (27.1%) |  |
| 5-6 | 16 (9.0%) | 10 (14.3%) |  |
| >6 | 9 (5.1%) | 9 (12.9%) |  |
| How many potential tutors are available in your institution in this specific area you would like to pursue? |  |  | 0.300 |
| 0 | 11 (6.2%) | 3 (4.3%) |  |
| 1 | 40 (22.6%) | 11 (15.7%) |  |
| 2 | 37 (20.9%) | 11 (15.7%) |  |
| >2 | 75 (42.4%) | 36 (51.4%) |  |
| Did the tutor routinely schedule scientific meetings and/or journal clubs? |  |  | 0.621 |
| Yes | 82 (46.3%) | 35 (50.0%) |  |
| No | 74 (41.8%) | 28 (40.0%) |  |
| Did the tutor set up a hierarchical structure in order to assure a tutorial program to fellows? |  |  | 0.503 |
| Yes | 92 (52.0%) | 42 (60.0%) |  |
| No | 63 (35.6%) | 20 (28.6%) |  |
| Do the scientists/researchers which are colleagues of the tutor collaborate to train the fellows? |  |  | 0.271 |
| Yes | 119 (67.2%) | 55 (78.6%) |  |
| No | 39 (22.0%) | 8 (11.4%) |  |
| Is it an exciting and pleasurable place to work? |  |  | 0.156 |
| Yes | 123 (69.5%) | 57 (81.4%) |  |
| No | 48 (27.1%) | 12 (17.1%) |  |
| Do tutors treat fellows sensibly and professionally? |  |  | 0.577 |
| Yes | 128 (72.3%) | 51 (72.9%) |  |
| No | 34 (19.2%) | 12 (17.1%) |  |
| Has each fellow an adequate working space with fully available equipment and supplies? |  |  | 0.594 |
| Yes | 83 (46.9%) | 36 (51.4%) |  |
| No | 76 (42.9%) | 30 (42.9%) |  |
| Is there opportunity to establish collaborations with other research groups? |  |  | 0.021 |
| Yes | 131 (74.0%) | 64 (91.4%) |  |
| No | 33 (18.6%) | 3 (4.3%) |  |
| Can the tutor send fellows abroad for training? |  |  | 0.189 |
| Yes | 118 (66.7%) | 50 (71.4%) |  |
| No | 35 (19.8%) | 14 (20.0%) |  |
| What would be your geographic region of choice to temporary continue your training? |  |  | 0.298 |
| North America | 57 (32.2%) | 29 (41.4%) |  |
| Central and South America | 0 | 1 (1.4%) |  |
| Northern and Continental Europe | 65 (36.7%) | 20 (28.6%) |  |
| Mediterranean countries | 53 (29.9%) | 19 (27.1%) |  |
| Asia and Pacific | 2 (1.1%) | 1 (1.4%) |  |
| Has the tutor the opportunity to provide scholarship to fellows? |  |  | 0.006 |
| Yes | 75 (42.4%) | 44 (62.9%) |  |
| No | 84 (47.5%) | 17 (24.3%) |  |
| Is the tutor willing to foster the fellow independence? |  |  | 0.066 |
| Yes | 126 (71.2%) | 58 (82.9%) |  |
| No | 31 (17.5%) | 4 (5.7%) |  |
| Does the tutor train fellows in writing scholarly papers? |  |  | 0.001 |
| Yes | 85 (48.0%) | 53 (75.7%) |  |
| No | 76 (42.9%) | 13 (18.6%) |  |
| Does the tutor train fellows in writing research grants? |  |  | <0.001 |
| Yes | 50 (28.2%) | 40 (57.1%) |  |
| No | 107 (60.5%) | 23 (32.9%) |  |
| Does the tutor really help fellows in finding an academic position or an appropriate professional employment? |  |  | 0.055 |
| Yes | 80 (45.2%) | 40 (57.1%) |  |
| No | 70 (39.5%) | 15 (21.4%) |  |
| If you had to do it all over again, would you choose to pursue research/clinical training in this same institution? |  |  | 0.267 |
| Yes | 114 (64.4%) | 54 (77.1%) |  |
| No | 51 (28.8%) | 13 (18.6%) |  |
