# Supplementary material for: An International Survey on Taking Up a Career in Cardiovascular Research: Opportunities and Biases toward Would-Be Physician-Scientists
Source: PLoS One. 2015 Jul 17;10(7):e0131900. doi: 10.1371/journal.pone.0131900 (PMC4506064; doi:10.1371/journal.pone.0131900)
Supplement: S5 Table — (DOC) [file pone.0131900.s005.doc]

**Table S5.** Multivariable analysis for selected responses.*

|  | **Age ≤30** | **Female gender** | **North America/Northern or Continental Europe area of origin** | **Training phase** | **Full-time researcher status** |
| --- | --- | --- | --- | --- | --- |
| More than 4 potential areas/fields of research concerning cardiovascular sciences offered | P>0.20 | P>0.20 | P>0.20 | P>0.20 | OR=1.68 (0.97-2.89), p=0.064 |
| Field of research concerning cardiovascular sciences you have pursued was first preference | P>0.20 | OR=0.40 (0.22-0.71), p=0.002 | OR=1.98 (1.04-3.77), p=0.037 | P>0.20 | P>0.20 |
| Tutor available for consultation >4 times per week. | P>0.20 | OR=0.39 (0.17-0.90), p=0.027 | P>0.20 | OR=2.03 (0.86-4.81), p=0.106 | OR=2.73 (1.19-6.30), p=0.018 |
| Scientists/researchers which are colleagues of the tutor collaborate to train the fellows | P>0.20 | OR=0.58 (0.32-1.05), p=0.072 | OR=1.71 (0.88-3.32), p=0.113 | P>0.20 | P>0.20 |
| Institution is an exciting and pleasurable place to work | OR=0.66 (0.35-1.23), p=0.187 | P>0.20 | OR=4.39 (1.95-9.86), p<0.001 | P>0.20 | P>0.20 |
| Tutors treat fellows sensibly and professionally | P>0.20 | OR=0.64 (0.34-1.21), p=0.173 | OR=1.97 (0.96-4.03), p=0.065 | OR=1.81 (0.98-3.34), p=0.058 | P>0.20 |
| Each fellow has an adequate working space with fully available equipment and supplies | P>0.20 | P>0.20 | OR=4.61 (2.49-8.52), p<0.001 | P>0.20 | P>0.20 |
| There is opportunity to establish collaborations with other research groups | P>0.20 | P>0.20 | P>0.20 | OR=0.36 (0.17-0.77), p=0.009 | P>0.20 |
| North America/Northern or Continental Europe is geographic region of choice to continue training | P>0.20 | P>0.20 | OR=4.09 (1.90-8.83), p<0.001 | P>0.20 | P>0.20 |
| Tutor trains fellows in writing research grants | P>0.20 | P>0.20 | OR=1.55 (0.87-2.74), p=0.135 | P>0.20 | OR=1.94 (1.11-3.37), p=0.020 |
| The tutor really helps fellows in finding an academic position or an appropriate professional employment | P>0.20 | OR=0.37 (0.20-0.67), p=0.001 | OR=2.06 (1.02-4.16), p=0.043 | P>0.20 | P>0.20 |
| If having to do it all over again, respondent would choose to pursue research/clinical training in this same institution | P>0.20 | OR=0.37 (0.20-0.67), p=0.001 | OR=2.06 (1.02-4.16), p=0.043 | P>0.20 | P>0.20 |

*stemming from a backward stepwise logistic regression model (P of removal 0.20), including age, gender, geographic area of origin of respondents, and training phase, and full-time researchers status, as independent variables, and reporting odds ratios (OR) with 95% confidence intervals and corresponding p values
