# Supplementary material for: An International Survey on Taking Up a Career in Cardiovascular Research: Opportunities and Biases toward Would-Be Physician-Scientists
Source: PLoS One. 2015 Jul 17;10(7):e0131900. doi: 10.1371/journal.pone.0131900 (PMC4506064; doi:10.1371/journal.pone.0131900)
Supplement: S1 Data — (DOC) [file pone.0131900.s006.doc]

**Data S1. Search strategy.**

Scholarly papers used to generate the questionnaire were searched in MEDLINE/PubMed up to December 31, 2013 according to the following string (("heart"[MeSH Terms] OR "heart"[All Fields] OR "cardiac"[All Fields]) OR ("cardiovascular system"[MeSH Terms] OR ("cardiovascular"[All Fields] AND "system"[All Fields]) OR "cardiovascular system"[All Fields] OR "cardiovascular"[All Fields]) OR ("blood vessels"[MeSH Terms] OR ("blood"[All Fields] AND "vessels"[All Fields]) OR "blood vessels"[All Fields] OR "vascular"[All Fields])) AND (mentor[All Fields] OR mentored[All Fields] OR mentoring[All Fields] OR mentorship[All Fields]) AND ("0001/01/01"[PDAT] : "2013/12/31"[PDAT]), eventually retrieving a total of 323 citations.

Authors having recently published a cardiovascular paper and thus suitable for invitation to participate to the survey and their corresponding email addresses were searched in MEDLINE/PubMed up to May 31, 2014, according to the following string (("heart"[MeSH Terms] OR "heart"[All Fields] OR "cardiac"[All Fields]) OR ("cardiovascular system"[MeSH Terms] OR ("cardiovascular"[All Fields] AND "system"[All Fields]) OR "cardiovascular system"[All Fields] OR "cardiovascular"[All Fields])) AND ("2010/01/01"[PDAT] : "2014/05/31"[PDAT]), eventually retrieving a total of 370,842 citations.
