# Supplementary material for: An International Survey on Taking Up a Career in Cardiovascular Research: Opportunities and Biases toward Would-Be Physician-Scientists
Source: PLoS One. 2015 Jul 17;10(7):e0131900. doi: 10.1371/journal.pone.0131900 (PMC4506064; doi:10.1371/journal.pone.0131900)
Supplement: S2 Data — (DOC) [file pone.0131900.s007.doc]

**Data S2. Questionnaire.**

1) How many potential areas/fields of research concerning cardiovascular sciences interest did your institution offer?

- 1-2
- 3-4
- 5-6
- >6

2) The field of research concerning cardiovascular sciences you have pursued was your first preference?

- yes
- no
- I have not yet begun any specific research project

3) How many potential tutors are available your institution in this specific area you would like to pursue?

- 1-2
- 3-4
- 5-6
- >6
- I have not yet made up my mind on any specific research project

4) How many times in a week is the tutor is available for consultation?

- 0
- 1
- 2
- >2
- not applicable

5) Did the tutor routinely schedule scientific meetings and/or Journal Clubs?

- yes
- no
- not applicable

6) Did the tutor set up a hierarchical structure in order to assure a tutorial program to fellows?

- yes
- no
- not applicable

7) Do the scientists/researchers which are colleagues of the tutor collaborate to train the fellows?

- yes
- no
- not applicable

8) Is it an exciting and pleasurable place to work?

- yes
- no

9) Do tutors treat fellow professionally and with cultural sensitivity?

- yes
- no
- not applicable

10) Does each fellow have an adequate working space with equipment and supplies full available?

- yes
- no
- not applicable

11) Is there opportunity to establish collaborations with other research groups?

- yes
- no
- not applicable

12) Has the mentor the opportunity to send fellows for training abroad?

- yes
- no
- not applicable

13) What would you your country of choice to temporary continue your training? _____________

14) Has the tutor the opportunity to provide scholarship (obtained from research grants) to fellow?

- yes
- no
- not applicable

15) Is the tutor interested in make possible fellow's transition to independence?

- yes
- no
- not applicable

16) Does the tutor train fellows in writing papers?

- yes
- no
- not applicable

17) Does the tutor train fellows in writing research grants?

- yes
- no
- not applicable

18) Does the tutor really help fellows in finding an academic position or an appropriate professional employment?

- yes
- no
- not applicable

19) If you had to do it all over again, would you choose to pursue research/clinical training in this institution?

- yes
- no
- not applicable

20) What is your gender?

- male
- female

21) What is your age?

- <25 years
- 25-30 years
- 31-35 years
- 36-40 years
- 41-45 years
- >45 years

22) In which country is the institution you referred to? ___________________________________

23) What is your current position?

- undergraduate student
- graduate student
- post-graduate student
- resident
- fellow
- assistant Professor
- associate Professor
- full Professor
- consultant
- other (specify ________________________________________________________________)
