# Supplementary material for: An International Survey on Taking Up a Career in Cardiovascular Research: Opportunities and Biases toward Would-Be Physician-Scientists
Source: PLoS One. 2015 Jul 17;10(7):e0131900. doi: 10.1371/journal.pone.0131900 (PMC4506064; doi:10.1371/journal.pone.0131900)
Supplement: S3 Data — (PDF) [file pone.0131900.s008.pdf]

VERBALE DELLA SEDUTA DEL CONSIGLIO DI DIPARTIMENTO DI SCIENZE E BIOTECNOLOGIE MEDICO-CHIRURGICHE

Il giorno 30 Gennaio 2014 alle ore 14.30 si è riunito, ai sensi dell'art. 84 del D.P.R. 382/80, nell'Aula 7 della sede di C.so della Repubblica, 79, il Consiglio del Dipartimento di Scienze e Biotecnologie Medico-Chirurgiche dell'Università degli Studi di Roma "La Sapienza" per discutere il seguente:

ORDINE DEL GIORNO

Professori di ruolo di I e II fascia, Ricercatori, Rappresentanti del personale non docente, Rappresentanti degli studenti:

1. Comunicazioni
2. Approvazione verbale della seduta precedente
3. Personale docente
   - Chiamata inquadramento idonei, mobilità, verifica triennale, conferma in ruolo, congedi, nulla osta ecc.
   - Programmazione reclutamento 2013-2015.
4. Personale non docente
5. Affari Contabili e patrimoniali
   - approvazione bilancio di esercizio 2013; rendiconto in contabilità finanziaria.
6. Assegni di Ricerca e Borse di Studio
7. Affidamenti di incarichi per consulenze professionali, prestazioni di lavoro autonomo di natura occasionale e coordinata e continuativa
8. Frequentatori scientifici
9. Master. Corsi Alta formazione
10. Richieste di finanziamento:
    - cofinanziamento a sostegno di Accordi Interuniversitari internazionali di collaborazione culturale e scientifica (anno 2014)
    - cofinanziamento per lo svolgimento di attività di ricerca congiunta da parte di professori visitatori stranieri (anno 2014)
11. Contratti e Convenzioni
12. Varie ed eventuali

	COGNOME	NOME	P	A	AG
PO	ALVARO	DOMENICO	x		
PO	BIZZARRI	FEDERICO	x		
PO	DELLA ROCCA	CARLO	x		
PO	DI CARLO	ANGELINA		x	
PO	FRATI	GIACOMO	x		
PO	GALLO	ANDREA	x		
PO	MACCARI	STEFANIA			x
PO	MARODER	MARELLA	x		
PO	NERVI	CLARA	x		

PO	PIERELLI	FRANCESCO		x	
PO	RAGONA	GIUSEPPE	x		
PO	STAGNITTI	FRANCO			x
PO	TOMAO	SILVERIO			x
PA	ALESSANDRI	CESARE	x		
PA	BUSINARO	RITA	x		
PA	CALOGERO	ANTONELLA	x		
PA	CARBONE	ANTONIO	x		
PA	CENTANNI	MARCO		x	
PA	DE BIASE	DANIELA			x
PA	GIACOMINI	PATRIZIA			x
PA	IULIANO	LUIGI			x
PA	LENDARO	EUGENIO	x		
PA	MATTIA	CONSALVO	x		
PA	PAOLINI	SERGIO		x	
PA	PAROLI	MARINO	x		
PA	PASCALE	ESTERINA	x		
PA	RAIMONDI	GIANFRANCO			x
PA	ROMEO	GIOVANNA	x		
PA	SALVATI	MAURIZIO	x		
PA	SILECCHIA	GIANFRANCO	x		
PA	SPADEA	LEOPOLDO			x
PA	STRURNIOLO	MARIA GABRIELLA			x

RU	BERSANI	GIUSEPPE			x
RU	BIONDI ZOCCAI	GIUSEPPE			x
RU	CAMILLO	ELEONORA	x		
RU	CAPOCCI	MAURO	x		
RU	CARAMANICO	LUCIANO			x
RTD	CARDINALE	VINCENZO	x		
RU	CARINI	LUISA	x		
RU	CASALI	CARLO	x		
RU	CAVALLARO	GIUSEPPE			x
RU	CAVARRETTA	ELENA	x		
RU	CERQUIGLINI	ANTONELLA	x		
RU	CHECQUOLO	SAULA	x		
RU	CHIMENTI	ISOTTA	x		
RU	CODACCI PISANELLI	GIOVANNI			x
RU	COLUZZI	FLAMINIA	x		
RTD	CRISTALLI	MARIA PAOLA	x		

RU	CURRA'	ANTONIO			x
RU	DE FALCO	ELENA	x		
RU	DE PASCALE	ADELE		x	
RU	DI CRISTOFANO	CLAUDIO	x		
RU	FIERMONTE	GIANCARLO			x
RU	IARICCI	GIAN PIERO			x
RU	MARULLO	ANTONINO			x
RU	MISCUSI	MASSIMO	x		
RU	MOSCHETTA	ALFREDO		x	
RU	PASTORE	ANTONIO LUIGI	x		
RU	PAURI	FLAVIA			x
RU	PETROZZA	VINCENZO	x		
RU	PONTI	DONATELLA	x		
RU	POTENZA	CONCETTA			x
RU	POZZESSERE	GIUSEPPE			x
RU	SERRAO	MARIANO		x	
RU	SKROZA	NEVENA	x		
RU	SPAZIANI	ERASMO		x	
RU	VALENTE	GABRIELE		x	
RU	VINGOLO	ENZO MARIA		x	
**RAPPRESENTANTI STUDENTI, SPECIALIZZANDI, DOTTORANDI**					
	CORTESE	FRANCESCA		x	
	DEL DUCA	SUSANNA C.		x	
	LE ROSE	MARGHERITA		x	
	PAPA	ANSELMO		x	
	SILVESTRI	LUIGI		x	
**SEGRETARIO AMMINISTRATIVO**					
	FORTE	MARIA ROMANA	x		
	ALIBRANDI	RITA	x		
	DI STEFANO	GIUSEPPE		x	
	FODERARO	MARIA E.	x		
	MARRONE	RODOLFO V.	x		
	MASTROBUONO BATTISTI	PIETRO ANGELO	x		
	PULCRANO	MARIA		x	

Presiede il Direttore del Dipartimento Prof.ssa Marella Maroder, esercita le funzioni di Segretario il Segretario Amministrativo la dottoressa Maria Romana Forte.

Alle ore 14.35, constatata la presenza del numero legale, il Direttore apre la seduta.

1. **Comunicazioni**

Il Direttore illustra il regolamento per le elezioni per il rinnovo dei rappresentanti del personale docente e ricercatore e dei rappresentanti degli studenti nella Giunta di Facoltà che si svolgeranno il 28 Maggio 2014.

2. **Approvazione verbale della seduta precedente**

Il Direttore chiede al Consiglio se vi siano osservazioni sul Verbale del Consiglio di Dipartimento del 21 Novembre 2013. Non essendovene, ne mette a votazione l'approvazione.
Il Consiglio approva il Verbale all'unanimità.

3. **Personale docente**

- Chiamata inquadramento idonei, mobilità, verifica triennale, conferma in ruolo, congedi, nulla osta ecc.
Non sono pervenuti argomenti da trattare

- Programmazione reclutamento 2013-2015.

Il Direttore illustra quanto deliberato dal Senato Accademico e dal Consiglio di Amministrazione circa l'utilizzo delle risorse di cui alla programmazione del fabbisogno di personale 2014-2015 e ricorda che i Dipartimenti e le Facoltà sono chiamati a formulare proposte sulla destinazione di tali risorse, che verranno assegnate sulla base delle carenze didattiche, della collocazione dei Dipartimenti nella VQR in base ai terzili Sapienza e per motivate esigenze speciali relative a ricerca e didattica. Tali risorse saranno utilizzabili per upgrading di professori di I e II fascia (a riserva di partecipazione per personale della Sapienza) o per reclutamento dall'esterno e per reclutamento di ricercatori a tempo determinato di tipo B. Il Direttore informa inoltre il Consiglio che i criteri di ripartizione delle risorse sono ancora oggetto di discussione negli Organi Collegiali dell'Ateneo.

Dopo ampia discussione, il Consiglio delibera le seguenti proposte minime di attribuzione delle risorse:

Upgrading di Professore di I fascia

2014	
SC 06/C1	SSD MED/18
SC 06/N1	
2015	
SC 06/E3	SSD MED/27
SC 05/E1	SSD BIO/10

Upgrading di Professore di II fascia

2014	
SC 02/B3	SSD FIS/07
SC 06/D1	SSD MED/11
SC 06/C1	SSD MED/18
SC 06/D5	SSD MED/25
SC 06/D6	SSD MED/26
SC 06/E3	SSD MED/27
SC 06/L1	SSD MED/41
2015	
SC 06/C1	SSD MED/18

SC 06/D6	SSD MED/26
SC 06/N1	
RTD B	
2014	
SC 06/N1	
2015	
SC 06/D4	SSD MED/12
SC 05/H1	SSD BIO/16
SC 05/H2	SSD BIO/17

e delega il Direttore ad operare eventuali integrazioni alle richieste di personale che ritenga opportune ed a redigere la programmazione del Dipartimento in tutte le sue parti.

Approvato all'unanimità seduta stante.

4. Personale non docente

Non sono pervenuti argomenti da trattare

5. Affari Contabili e patrimoniali

- approvazione bilancio di esercizio 2013; rendiconto in contabilità finanziaria.

Il Consiglio, udita la relazione del Segretario Amministrativo e del Direttore, approva all'unanimità il Bilancio Consuntivo 2013, con i relativi documenti.
Letto ed approvato seduta stante.

- Il Consiglio, vista la richiesta del Prof. Marino Paroli, esprime parere favorevole alla accettazione della donazione, da parte di AbbVie srl, di una sonda ecografica Esaote LA523, necessaria alle attività di ricerca condotte nel Dipartimento di Scienze e Biotecnologie Medico Chirurgiche, per un importo di € 5600+IVA.
Letto e approvato seduta stante per la parte dispositiva.

- Il Consiglio, vista la richiesta della Prof.ssa Potenza, esprime parere favorevole alla accettazione dell'erogazione liberale di € 23.100,00 messa a disposizione dalla AvvVie srl, destinata al finanziamento di un assegno di ricerca della durata di un anno, dal titolo "Nuovo modello organizzativo e gestionale personalizzato per il paziente psoriasico complicato: creazione di una unità psoriasica",
da attribuire ad un laureato in Medicina e Chirurgia con Specializzazione in Dermatologia e Venereologia, che non usufruisca di altere borse di studio.
Letto e approvato seduta stante per la parte dispositiva.

- Il Consiglio, vista la richiesta del Prof. Carbone, esprime parere favorevole alla accettazione dell'erogazione liberale di € 2.500,00, messa a disposizione dalla Dornier MedTech Italia srl, a sostegno delle attività del Master Universitario di II Livello in "Innovazioni tecnologiche in chirurgia laparoscopia avanzata".
Letto e approvato seduta stante per la parte dispositiva.

- Il Consiglio, vista la richiesta del Prof. Carbone, esprime parere favorevole alla accettazione dell'erogazione liberale di € 5.000,00, messa a disposizione dalla B-Braun Milano SPA, a potenziamento delle attività scientifiche del Dipartimento.
Letto e approvato seduta stante per la parte dispositiva.

- Il Consiglio, vista la richiesta del Direttore, esprime parere favorevole alla risoluzione dell'accordo stipulato con Pfizer Italia dal titolo "Nf-kB Expression in Peripheral Lymphocytes and Psoriatic Skin, Before and After Treatment With Etanercept", responsabile Prof.ssa Potenza, causa criticità riscontrate nell'iter di approvazione da parte del Comitato Etico della ASL di Latina. Il Consiglio approva, altresi, la emissione di un ordinativo di spesa pari alla somma già versata dalla Pfizer Italia a titolo di acconto per la conduzione dello studio.

Letto e approvato seduta stante per la parte dispositiva.

- Il Consiglio, vista la richiesta del Prof. Alvaro, esprime parere favorevole alla richiesta di disinventario del Computer HP Compaq dx2400 N. Inv. 0928 in quanto non più funzionante.

Letto e approvato seduta stante per la parte dispositiva.

6. Assegni di Ricerca e Borse di Studio

- Il Consiglio, vista la richiesta del Responsabile scientifico Prof.ssa Clara Nervi, vista la relazione scientifica presentata, preso atto della necessità di non interrompere la conduzione della ricerca e in considerazione della programmazione a medio termine dell'attività, approva il rinnovo della borsa di studio della Dott.ssa Stefania Pisanò, dal titolo "Ruolo dei Nuclear Factor I nell'ematopoiesi normale e patologica", la cui spesa pari ad € 14.000,00 (oltre alle spese assicurative) graverà sul finanziamento Ricerca Scientifica 2010 e, in parte, su Fondi AIRC IG11949, titolare Prof.ssa Nervi.

Letto e approvato seduta stante per la parte dispositiva.

- Il Consiglio, vista la richiesta della Prof.ssa Potenza, approva la pubblicazione di 2 bandi per assegni di ricerca SSD MED/35 :

1) Bando per assegno di ricerca Cat.B, tipo I, annuale, su fondi Abbvie srl, dal titolo "Nuovo modello organizzativo e gestionale personalizzato per il paziente psoriasico complicato: creazione di una Unit psoriasica". Commissione giudicatrice proposta: Prof.ri Della Rocca, Potenza, Skroza, Raimondi, Calogero.
2) Bando per assegno di ricerca Cat B, tipo I, biennale, su fondi Pharmaroma, dal Titolo: "la medicina di genere nell'acne: un trattamento innovativo diversificato". Commissione giudicatrice proposta: Prof.ri Della Rocca, Potenza, Skroza, Ragona, Calogero.

Letto e approvato seduta stante per la parte dispositiva.

- Il Consiglio, vista la richiesta del Prof. Ragona, approva la pubblicazione di 1 bando per assegno di ricerca SSD MED/04:

1) Bando per assegno di ricerca Cat.B, tipo II, annuale, su fondi Ricerca 2013, dal titolo "Azione ormonale tiroidea, infertilità femminile e atresia follicolare, apoptosi e sopravvivenza cellulare". Commissione giudicatrice proposta: Prof.ri Centanni, Ragona, Gargiulo.

Letto e approvato seduta stante per la parte dispositiva.

- Il Consiglio, vista la richiesta del Direttore, ratifica i decreti n. 6,7,8 e 12 del 2014, con i quali il Direttore ha nominato le Commissioni giudicatrici per il conferimento di assegni di ricerca e borse di studio.

Letto e approvato seduta stante per la parte dispositiva.

7. Affidamenti di incarichi per consulenze professionali, prestazioni di lavoro autonomo di natura occasionale e coordinata e continuativa.

Non sono pervenuti argomenti da trattare.

8. Frequentatori scientifici

- Il Direttore sottopone all'approvazione del Consiglio la richiesta con la quale la Prof.ssa Businaro, chiede di ammettere come frequentatore del laboratorio di cui è responsabile, la Dott. ssa Silvia Carlomagno. A tal fine, specifica che la Dott.ssa Carlomagno ha stipulato regolare polizza assicurativa, di cui ha presentato copia.
Il Consiglio approva all'unanimità.

- Il Direttore, sottopone a ratifica l'ammissione a frequentare il Dipartimento in qualità di fellow, a partire dal prossimo Ottobre, del Dr. Samer Al Rawashdah per approfondimento degli studi in materia di oncourologia, sotto la supervisione del Prof. Carbone. A tal fine, specifica che nessun onere sarà a carico del Dipartimento e che tutte le spese relative al viaggio ed alla permanenza del Dr. Al Rawashdah saranno interamente sostenute dall'università di provenienza e che, come richiesto dall'Ateneo, il Dr. Al Rawashdah stipulerà regolare polizza assicurativa, di cui presenterà copia.
Il Consiglio approva all'unanimità.

9. Master. Corsi Alta formazione

Non sono pervenuti argomenti da trattare

10. Richieste di finanziamento

- cofinanziamento a sostegno di Accordi Interuniversitari internazionali di collaborazione culturale e scientifica (anni 2013-14)

- Il Consiglio, su richiesta della Prof. ssa Adele De Pascale, responsabile della domanda di Finanziamento per Accordi Internazionali, A.F. 2014 – Bando D.R. 741, Accordo con Universidad San Sebastian (Cile), delibera l'assunzione di impegno del Dipartimento alla copertura finanziaria del 10% del contributo totale richiesto, a titolo di cofinanziamento (per un massimo di € 500).
Letto e approvato seduta stante per la parte dispositiva.

- Il Consiglio, su richiesta del Prof. Giuseppe Bersani, responsabile della domanda di Finanziamento per Accordi Internazionali, A.F. 2014 – Bando D.R. 741, Accordo con Universidad de la Habana (Cuba), delibera l'assunzione di impegno del Dipartimento alla copertura finanziaria del 10% del contributo totale richiesto, a titolo di cofinanziamento (per un massimo di € 500).
Letto e approvato seduta stante per la parte dispositiva.

- cofinanziamento per lo svolgimento di attività di ricerca congiunta da parte di professori visitatori stranieri (anni 2013-14)

- Il Consiglio, su richiesta della Prof. Rita Businaro, responsabile della domanda di Finanziamento per Professori visitatori per attività di Ricerca anno 2013 per l'invito del Prof. Trevor Archer, Professor presso la Goteberg University (Svezia), delibera l'assunzione di impegno del Dipartimento alla copertura finanziaria del 10% del contributo totale richiesto, a titolo di cofinanziamento.
Letto e approvato seduta stante per la parte dispositiva.

11. Contratti e Convenzioni

- Il Consiglio, vista la richiesta dell'del Prof. Carbone, esprime parere favorevole alla stipula di un contratto tra il Dipartimento di Scienze e Biotecnologie Medico Chirurgiche e l'azienda Astellas Pharma Europe B.V., per il tramite del suo rappresentante autorizzato INC Research Italia srl, in merito allo svolgimento del protocollo clinico 178 CL-102. Il suddetto protocollo sarà effettuato nell'ambito dell'attività di ricerca del gruppo Urologico del Dipartimento, anche in ragione della propria attività

clinica svolta all'interno della Unità Operativa Complessa di Urologia a direzione universitaria dell'AUSL di latina, presso il polo ospedaliero integrato "AUSL LATINA – Padiglione ICOT".
Letto e approvato seduta stante per la parte dispositiva.

- Il Consiglio, vista la richiesta della Prof.ssa Potenza, esprime parere favorevole alla stipula di un contratto tra il Dipartimento di Scienze e Biotecnologie Medico Chirurgiche e la Pharmaroma 2005, avente ad oggetto lo studio osservazionale "La Medicina di genere nell'acne: un trattamento innovativo diversificato", in attesa di approvazione dal Comitato Etico Lazio 2. Per la conduzione di tale studio, la Pharmaroma 2005 verserà al Dipartimento € 77000 IVA inclusa. Il suddetto protocollo sarà effettuato nell'ambito dell'attività di ricerca del gruppo di Dermatologi del Dipartimento, anche in ragione della propria attività clinica svolta all'interno della Unità Operativa di Dermatologia a direzione universitaria dell'AUSL di Latina, presso l'Ospedale "Fiorini" di Terracina.

Letto e approvato seduta stante per la parte dispositiva.

- Il Consiglio, vista la richiesta del Dott. Marullo, esprime parere favorevole alla sottoscrizione di un Protocollo esecutivo per ricerca in Cardiochirurgia, Cardiologie e Biotecnologie con l'Università of SS Ciril and Methodius in Skopje (Macedonia)

Letto e approvato seduta stante per la parte dispositiva.

12. Varie ed eventuali

- Il Consiglio, su proposta del Direttore, nomina, quali componenti del seggio elettorale per le elezioni per il rinnovo dei rappresentanti del personale docente e ricercatore e dei rappresentanti degli studenti nella Giunta di Facoltà, ubicato presso il Dipartimento, i seguenti docenti: Nervi, Chimenti, Lendaro. Membro supplente: De Falco.
  Letto e approvato seduta stante per la parte dispositiva.

- Il Consiglio su richiesta del Prof. Giacomo Frati autorizza lo svolgimento della Survey-questionario on-line dal titolo "Engaging into a career in cardiovascular research" per fini scientifici e didattico-divulgativi.

- Il Consiglio, vista la richiesta del Direttore, nomina, quali componenti della Commissione Benessere Animale, la Prof.ssa Calogero, la Prof.ssa Businaro, il Dott.Petrozza.

Letto e approvato seduta stante per la parte dispositiva.

- Il Consiglio, vista la richiesta del Direttore, nomina, quali componenti della Commissione VQR, la Prof.ssa Nervi, il Dott. Di Cristofano e la Sig.ra Meschini.

Alle ore 16.30, esauriti gli argomenti all'ordine del giorno, termina il Consiglio di Dipartimento aperto a tutte le componenti. La seduta è tolta.

Il Direttore del Dipartimento
(Prof. ssa Marella Maroder)

Marella Maroder
