# Supplementary material for: An International Survey on Taking Up a Career in Cardiovascular Research: Opportunities and Biases toward Would-Be Physician-Scientists
Source: PLoS One. 2015 Jul 17;10(7):e0131900. doi: 10.1371/journal.pone.0131900 (PMC4506064; doi:10.1371/journal.pone.0131900)
Supplement: S4 Data — (DOC) [file pone.0131900.s009.doc]

**Data S4. Invitation letter.**

Subject: Survey on mentoring in clinical research

Dear Colleague

I am writing to invite you to complete a brief survey on mentoring in cardiovascular research.

To participate just follow this link: www.cardiogroup.org/survey/career

Indeed, as part of a larger program to evaluate the quality of education programs in Western countries, we are conducting a survey entitled "Engaging into a career in cardiovascular research". The purpose of this work is to evaluate opportunities and perspectives for would-be physician-scientists.

Duration: You will be able to complete the survey in no more than 1-2 minutes.

Scope: The survey includes questions about your career history as well as your perceptions on training programs in cardiovascular research, the availability of opportunities that you have to develop and maintain friendships, and your perceptions of possibility of career in general.

Confidentiality: your responses will be kept completely confidential. We will NOT know your IP address when you respond to the Internet survey.

Risks or discomforts: There are no known risks if you decide to participate in this research study, nor are there any costs for participating in the study. Your participation is voluntary; you are free to withdraw your participation from this study at any time.

Notice: If you do not want to continue, you can simply discard this message or leave the survey website. If you do not click on the "submit" button at the end of the survey, your answers and participation will not be recorded. You also may choose to skip any questions that you do not wish to answer. If you click on the "submit" button at the end of the survey, you will be entered in the analysis set.

Thank you in advance for your participation to this important project. If you have any questions on the administration of the survey, please contact Dr. Mariangela Peruzzi at mariangela.peruzzi@uniroma1.it, Sapienza University of Rome, Rome, Italy.

Yours truly

Giacomo Frati, MD

Department of Medico-Surgical Sciences and Biotechnologies

Sapienza University of Rome

Rome

Italy
